# Evidence of Left Ventricular Cardiac Remodeling After 6 Weeks of Sprint Interval Training

**DOI:** 10.1111/sms.70007

**Published:** 2024-12-20

**Authors:** Lisa M. J. Eriksson, Kristofer Hedman, Meriam Åström‐Aneq, Eva Nylander, Karin Bouma, Mirko Mandić, Thomas Gustafsson, Eric Rullman

**Affiliations:** ^1^ Division of Clinical Physiology, Department of Laboratory Medicine Karolinska Institutet Stockholm Sweden; ^2^ Unit of Clinical Physiology Karolinska University Hospital Stockholm Sweden; ^3^ Department of Clinical Physiology, and Department of Health, Medicine and Caring Sciences Linköping University Linköping Sweden

**Keywords:** blood volume, bradycardia, cardiac function, echocardiography, high‐intensity exercise, hypervolemia

## Abstract

Sprint interval training (SIT) leads to similar improvements in maximal oxygen uptake (V̇O_2max_) and maximal cardiac output as previously reported for traditional endurance training, but the exercise‐induced effects on cardiac remodeling are still largely unknown. The aim of the current study was therefore to explore the effects of SIT on cardiac structure and function assessed by echocardiography in relation to, and controlling for, changes in both blood volume (BV) and heart rate (HR). Healthy men and women (*n* = 28) performed 6 weeks of SIT. V̇O_2max_ and total BV were measured, and echocardiography was performed before and after the intervention. There was a robust increase in BV (+7.1%; *p* < 0.001) and V̇O_2max_ (+10.1%; *p* < 0.001) and a decrease in resting HR (−3.9%; *p* = 0.013) following the SIT intervention. Machine‐learning‐based feature selection and univariate analysis indicated that several measures of left ventricular dimension (+14.3% LVEDV, *p* = 0.013; +17.4% LVESV, *p* = 0.018; +12.3% LVSV, *p* = 0.031), left ventricular diastolic function (MV_A_, MV_D‐slope_, MV_DT_), and left ventricular stroke volume (LVOT VTI) were altered by 6 weeks of SIT. When controlling for the exercise‐induced changes in BV and HR, left ventricular dimensions remained significantly changed. Our data indicate that several measures of cardiac function are likely only indirectly affected by SIT, driven by increased BV. However, the disproportionate increase in left ventricular size exceeds what can be explained by changes in BV and HR alone, indicating volume‐independent structural cardiac remodeling.

## 1 Introduction

The term “athlete's heart” refers to proportional increases in the end‐diastolic dimensions and volume of the left (LV) and right ventricle (RV) [1], LV wall thickness [1, 2], left atrium (LA) volume [3], and LV mass [1, 2, 4], together, contributing as an underlying basis for the exercise‐induced increase in maximal cardiac output [5]. Functional adaptations observed after traditional endurance training (TET) include improved LV compliance [6, 7] as well as a lower heart rate (HR) at rest and during submaximal exercise [5]. For sprint interval training (SIT), short‐term studies have reported changes in LV volume [8], LV mass, stroke volume [9] and LV systolic, and diastolic function [10] in addition to resting HR [9, 10], all of which are consistent with the cardiovascular adaptations seen in endurance athletes [5]. Research suggests that the characteristics of cardiovascular adaptations differ between TET and resistance training due to differences in hemodynamic demands and cardiac load during exercise [11]. However, despite the vast difference in hemodynamic stress between SIT and TET, changes in blood volume (BV) and maximal oxygen uptake (V̇O_2max_) have been reported to be similar [12, 13].

Increased BV is a possibly underestimated mechanism underlying cardiac remodeling to exercise training [14]. Exercise‐induced hypervolemia allows increased venous return and cardiac preload during exercise [15], which contributes to an increase in stroke volume [16] and a compensatory increase in parasympathetic activity in athletes [17, 18], leading to secondary bradycardia. This is supported by evidence that cardiac output and systemic oxygen demand at rest are generally unaffected by exercise [5]. Thus, one could speculate that the dimensional and functional changes in the heart may be manifested solely by BV expansion and bradycardia.

The aim of this study was to characterize the changes in cardiac dimensions and function induced by SIT and to investigate the extent to which changes in BV and resting HR could explain these changes. We hypothesized that the exercise‐induced changes in cardiac output parameters correlate with the expected increase in BV and decrease in HR.

## 2 Materials and Methods

### 2.1 Ethical Approval

All subjects were informed verbally and in writing about the study before providing written informed consent to participate. The study was approved by the Swedish Ethical Review Authority (reference 2017/2446–32; approved December 21, 2017).

### 2.2 Subjects

Subjects were included as part of a cohort in a previously published paper by Mandić et al. [13] and comprised 28 healthy subjects (12 women and 16 men; age, 27 ± 2 years; height, 176 ± 3.0 cm; body mass, 74.1 ± 4.3 kg; body mass index, 23.8 ± 1.0 kg/m^2^) undergoing a 6‐week SIT intervention, with pre‐ and post‐intervention measurements of BV, V̇O_2max_, and echocardiography. None of the subjects performed regular interval training or participated in any other structured exercise program. Subjects were instructed to refrain from any other physical activity or training for the duration of the study. Of the 28 subjects included in this data set, one subject was unable to perform the post‐intervention V̇O_2max_ test due to flu‐like symptoms, and one subject was unable to undergo a BV measurement because a peripheral venous catheter could not be placed. Subjects completed 98% of intended training sessions.

### 2.3 Training Intervention

The SIT intervention consisted of three supervised exercise sessions per week over a 6‐week period, as previously described [13]. Each session lasted 10 min and consisted of three consecutive 30‐s all‐out sprints performed on a mechanically braked cycle ergometer (Monark 894E, Varberg, Sweden) with a braking force equivalent to 7.5% of body weight. Subjects were instructed to pedal as fast as possible against the inertial resistance of the cycle ergometer. Braking force was applied manually when the maximum cadence was reached, and subjects were given strong verbal encouragement during each interval. Sprints were separated by 2 min of unloaded cycling. Before the first interval, the subjects completed a short, unloaded warm‐up (2.5 min). The last interval was followed by 2 min of unloaded cycling, which served as a cool‐down.

### 2.4 Pre‐ and Post‐Intervention Measurements

Pre‐ and post‐intervention procedures included the following: (i) determination of total BV, (ii) an incremental cycling test to determine V̇O_2max_, and (iii) an echocardiographic examination. All procedures were performed the week before and the week after the SIT intervention. Measurements were performed in the order described above and on 3 different days: Tuesdays, Thursdays, and Fridays. Height and weight were measured in conjunction with the BV determination.

The optimized carbon monoxide (CO) rebreathing method was used to determine total hemoglobin mass (tHb), from which BV and plasma volume (PV) were calculated as described previously [19]. Concisely, after 15 min rest, a venous blood sample was taken from the median cubital vein and immediately analyzed for baseline carboxyhemoglobin (%HbCO), hemoglobin concentration [Hb], and hematocrit (Htc) (ABL 800, Radiometer A/S, Copenhagen, Denmark). End‐tidal CO was measured at baseline and after rebreathing using a CO gas analyzer (Dräger, PAC 700, Lübeck, Germany). Throughout rebreathing, the same gas analyzer was used to check for CO leaks. Subjects inhaled and rebreathed a gas mixture of chemically pure (99.97%) CO (0.8 mL/kg) and medical oxygen (AGA, Stockholm, Sweden) for 2 min before disconnecting from the spirometer (Blood tec GmbH, Bayreuth, Germany). Two venous blood samples (1 mL) were collected, one before rebreathing and one 7 min after administration of CO. The samples were then analyzed in duplicate for %HbCO (ABL 800, Radiometer A/S, Copenhagen, Denmark). tHb was analyzed as described previously [20]. The coefficient of variation for this method in our laboratory is 1.0%–1.6%, which is in agreement with previous publications [21].

To determine V̇O_2max_ and maximal workload (W_max_), subjects performed an incremental cycling test to volitional fatigue on an electronically braked ergometer (Lode, Groningen, The Netherlands). Identical pre‐ and post‐intervention protocols were used, in which a warm‐up period of 5 min at 50 W preceded an increase in resistance of 1 W every 3 s (20 W/min) until subjects reached volitional fatigue. Using an online gas collection system (Vmax Encore 229, Carefusion, California), fractions of inhaled and exhaled O_2_ and CO_2_ were measured continuously and recorded as breath‐by‐breath values. V̇O_2max_ was determined as the highest 20 s average reached during the test. Criteria for a maximal test included a plateau in V̇O_2_ or achievement of all of the following criteria: respiratory exchange ratio (RER) > 1.15, an HR within 10 beats of the age‐related maximum, and volitional exhaustion quantified by a rating of perceived exertion ≥ 18.

The echocardiographic examination was performed by a licensed biomedical scientist using the Vivid E9 ultrasound system (General Electric, Horten, Norway) and a 4V2c phased‐array transducer. Subjects were examined in the left lateral recumbent position after 5 min supine rest, and resting HR was then determined at four timepoints during the examination. The echocardiography was performed according to the recommendations from the American Society of Echocardiography and the European Association of Cardiovascular Imaging [22]. Cardiac chamber quantification of LV and RV dimensions and systolic and diastolic parameters was performed by an experienced reader blinded to the time for the examination pre‐ or post‐SIT intervention. LV systolic function was assessed by calculating LV stroke volume (LVSV) and ejection fraction (EF) using the Simpson biplane method and global longitudinal strain (GLS) based on 2D speckle tracking in 4‐, 3‐, and 2‐chamber views. LA size, diastolic E/A ratio, mitral valve E‐wave deceleration time (MV_DT_), and E/e' (e' mean of the septal and lateral wall) were measured to determine diastolic LV function. RV systolic function included tricuspid plane systolic excursion (TAPSE) and longitudinal strain by speckle tracking. All measurements were performed according to current guidelines [22, 23]. LV mass was calculated as LV mass = 0.8 × 1.04 × [(IVSd + LVIDd + PWd)^3^—LVIDd^3^] + 0.6 g. Image analysis and measurements were performed offline using standard software (EchoPAC 202, GE Healthcare). Cardiac output at rest was calculated from the echocardiographic‐derived measurements of LVSV and resting HR, as Cardiac output = HR × LVSV. Dimensional and volumetric variables were analyzed as absolute data.

### 2.5 Statistics

To facilitate interpretation, the variables were categorized into LV morphological features, LV systolic parameters, LV diastolic parameters, and RV structure and function. Mutual dependence and correlation between the echocardiographic variables were tested by Pearson correlation in a correlation matrix analysis and by principal component analysis (PCA). Global and mutual correlations were analyzed with PCA using Factominer (version 2.4). Multivariate analysis techniques such as PCA are superior to the use of numerous univariate models for high‐dimensional data, especially in the presence of collinearities. In high‐dimensional spaces, univariate methods analyze each outcome independently and ignore relationships and correlations, which can lead to lower statistical power and potentially misleading results. PCA, on the other hand, looks at the entire set of variables simultaneously and identifies the underlying structure of the data by reducing the dimensionality. It captures variance in fewer dimensions and addresses collinearity by combining correlated variables into principal components. This not only reduces noise but also allows for a more comprehensive understanding of complex, interrelated data patterns than univariate methods that treat each variable in isolation. Based on the evidence of strong correlations between numerous variables from PCA and correlation analysis, orthogonal partial least squares discriminant analysis (OPLS‐DA) was applied using ropls (version 1.24.0).

OPLS‐DA provides a ranking of the importance of all the variables in their contribution to separate between pre‐ and post‐interventional time points. To determine which echocardiographic variables contributed significantly to the separation of pre‐ and post‐intervention measurements, where a *q*‐value of < 0.05 was considered a significant model (pre‐ vs post‐training) and a Variable Importance in Projection (VIP) ≥ 1 was considered a significant variable contribution. Significant variables were analyzed for significant (nominal *p* < 0.05) differences between pre‐ and post‐intervention with a mixed linear model with individual study participants as a random effect and resting HR, BV, or both as a fixed effect using the linear and nonlinear mixed effects model (version 3.1.153) in the R environment. All data are presented as mean ± 95% confidence interval.

## 3 Results

Physiological data at pre‐ and post‐intervention on body composition, hematology parameters, and physical performance are summarized in Table 1. No change in body composition was observed after the SIT intervention. Data on physical performance and BV have been reported previously [13]. In summary, V̇O_2max_ and W_max_ increased significantly after the SIT intervention, by 10.1% (*p* < 0.001) and 11.5% (*p* < 0.001), respectively. BV increased by 7.1% from baseline (*p* < 0.001) and cardiac output at rest increased by 8.4% (*p* = 0.04). Calculated LV mass was not significantly changed (139 ± 13 g and 141 ± 12 g; *p* = 0.69) following the SIT intervention. No change was observed in the peak HR, while the resting HR decreased by 3.9% from 68 ± 4 beats per minute at baseline to 65 ± 4 beats per minute (*p* = 0.013) after the SIT intervention. This significant difference in resting HR between pre‐ and post‐SIT measurements was observed throughout the echocardiographic protocol, regardless of the time spent in the left lateral recumbent position. No differences in delta V̇O_2max_ (*p* = 0.119), BV (*p* = 0.246), or resting HR (*p* = 0.909) were detected between men and women.

**TABLE 1:** Summary of physiological data before (pre) and after (post) the exercise intervention.

	SIT		
	Pre	Post	%Change
Body composition			
Weight (kg)	74.1 ± 4.3^§^	73.8 ± 4.2^§^	−0.4 ± 0.6
Height (cm)	176 ± 3	176 ± 3	0.0 ± 0.0
BMI (kg/m^2^)	23.8 ± 1.0^§^	23.7 ± 0.9^§^	−0.4 ± 0.6
Hematology			
tHb (g)	716 ± 63^§^	759 ± 69^§^	+6.0 ± 2.3***
PV (L)	3.38 ± 0.16^§^	3.66 ± 0.19^§^	+8.4 ± 2.6***
BV (L)	5.55 ± 0.31^§^	5.94 ± 0.36^§^	+7.1 ± 2.2***
Cardiorespiratory parameters			
V̇O_2max_ (L/min)	2.99 ± 0.31^§^	3.29 ± 0.34^§^	+10.1 ± 3.5***
RER_peak_	1.26 ± 0.03^§^	1.23 ± 0.02^§^	−1.9 ± 1.5*
HR_max_ (bpm)	193 ± 3^§^	193 ± 3^§^	−0.4 ± 0.6
Hemodynamic parameters			
HR_rest_ (bpm)	68 ± 4	65 ± 4	−3.9 ± 6.1*
Cardiac output (L/min)	4.01 ± 0.34^§^	4.34 ± 0.35^§^	+8.4 ± 8.2*

*Note:* Values are presented as mean ± 95% confidence interval, *N* = 28.

Abbreviations: BMI, body mass index; BV, blood volume; HR_max_, maximum heart rate; HR_rest_, resting heart rate; PV, plasma volume; RER_peak_, peak respiratory exchange ratio; tHb, total hemoglobin mass; V̇O_2max_, maximum oxygen uptake.

§ *N* = 27.

* *p* < 0.05.

*** *p* < 0.001.

**FIGURE 1:**
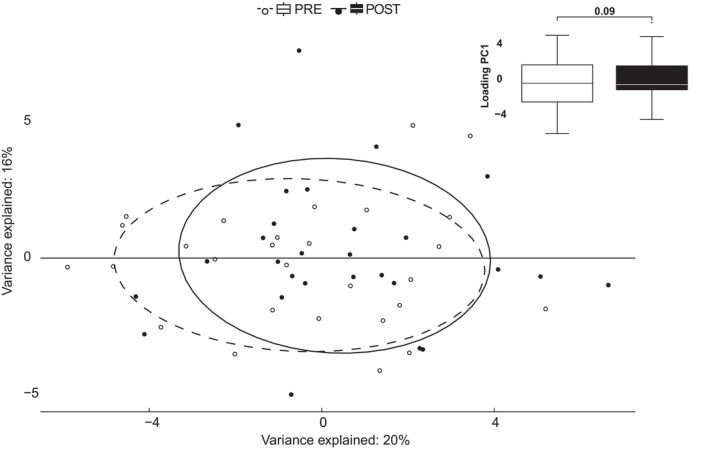
High degree of correlations among echocardiographic variables. Principal component analysis of the echocardiographic variables before (pre) and after (post) the exercise intervention shows a very high degree of shared variance and mutual correlations where approximately 35% of the total variation can be summarized by the first two components. In addition, there is a borderline statistically significant global shift indicating a difference between pre‐ and post‐variables. Based on data from all study participants (*N* = 28).

The large number of echocardiographic variables used to describe the more limited number of underlying physiological characteristics was evident in a high degree of mutually correlated variable pairs: Of the 36 investigated variables, 17 pairs were mutually correlated with a correlation coefficient > 0.68 (*p* < 0.001). Also, global shared variance on PCA was approximately 35% (Figure 1). PCA was performed on the separate classification groups which illustrated an even higher shared variance within each category: where the first two principal components accounted for 98% of the variability of variables describing LV morphological features (see Figure S1A), 50% of the variability of variables describing LV systolic function (see Figure S1B), 41% of the variability of variables describing LV diastolic function (see Figure S1C), and 86% of the variability of variables describing RV structure and function, respectively (see Figure S1D).

**FIGURE 2:**
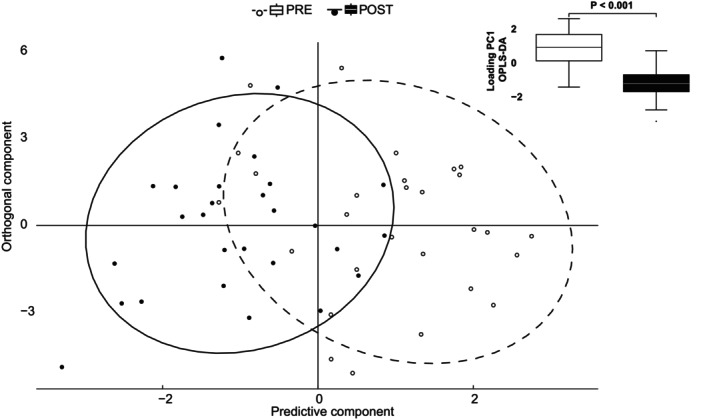
Six weeks of sprint interval training lead to significant changes in cardiac structure and function. Orthogonal Partial Least Square Discriminant Analysis (OPLS‐DA) showing the significant separation between the echocardiographic variables measured before (pre) and after (post) the exercise intervention. Based on data from all study participants (*N* = 28).

**FIGURE 3:**
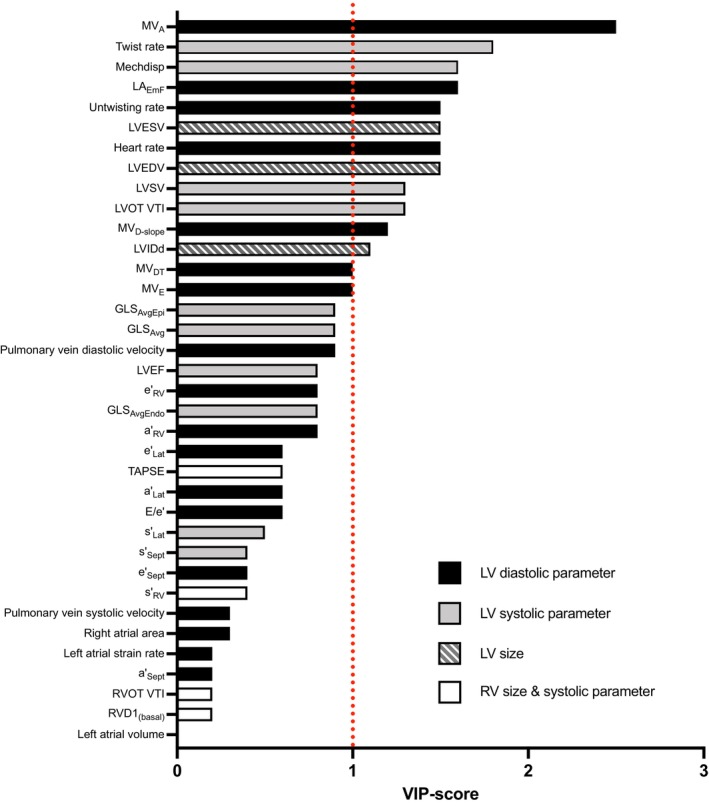
Thirteen measures of left ventricular structure and function changes significantly by 6 weeks of exercise training. The significance of the examined echocardiographic variables in distinguishing between before (pre) and after (post) exercise samples was elucidated through Variable Importance Projection (VIP) analysis. A VIP value equal to, or exceeding 1 (red‐dotted line), was deemed indicative of a substantial contribution to the analysis. Based on data from all study participants (*N* = 28). a'_Lat_, left ventricular lateral wall a'; a'_RV_, right ventricular free wall a'; a'_Sept_, left ventricular septal wall a'; E/e', mitral e‐wave velocity over average left ventricular e'; e'_Lat_, left ventricular lateral wall e'; e'_RV_, right ventricular free wall e'; e'_Sept_, left ventricular septal wall e'; GLS_Avg_, average global longitudinal strain; GLS_AvgEndo_, average endocardial global longitudinal strain; GLS_AvgEpi_, average epicardial global longitudinal strain; LA_EmF_, passive left atrial emptying fraction; LVEDV, left ventricular end‐diastolic volume; LVEF, left ventricular ejection fraction; LVESV, left ventricular end‐systolic volume; LVIDd, diastolic left ventricular inner diameter; LVOT VTI, left ventricular outflow tract velocity time integral; LVSV, left ventricular stroke volume; Mechdisp, mechanical dispersion; MV_A_, mitral valve A‐wave velocity; MV_D‐slope_, mitral valve deceleration slope; MV_DT_, mitral valve deceleration time; MV_E_, mitral valve E‐wave velocity; RVID1_(basal)_, right ventricular basal inner diameter; RVOT VTI, right ventricular outflow tract velocity time integral; s'_Lat_, left ventricular lateral wall s'; s'_RV_, right ventricular free wall s'; s'_Sept_, left ventricular septal wall s'; TAPSE, tricuspid annular plane systolic excursion.

An OPLS‐DA was applied to determine if there were any global differences between pre‐ and post‐intervention measurements in the echocardiographic variables and which individual echocardiographic measures that were most important for such separation. There was a significant global difference between pre‐ and post‐intervention observations (*p* < 0.001) which can be seen in Figure 2. Of the 36 echocardiographic variables tested, 14 (including resting HR) had a VIP score ≥ 1, indicating a significant contribution to the separation of the pre‐ and post‐intervention observations (Figure 3). All of the variables with a VIP ≥ 1 were measurements which could potentially be affected by both HR and BV. Subsequently, these variables were considered for further analysis using mixed linear models as shown in Table 2.

**TABLE 2:** Comprehensive summary of the echocardiographic variables assessed before (pre) and after (post) the exercise intervention. Significant changes in left ventricular volumes persist even when accounting for reduced resting heart rate and increased blood volume.

	SIT			*p‐*value controlled for change in		
	Pre	Post	%Change	Heart rate	Blood volume	Heart rate and blood volume
LV size						
LVEDV (mL)	95 ± 9^§^	109 ± 9^§^	+14.3 ± 8.3	0.004**	0.009**	0.013*
LVESV (mL)	36 ± 4^§^	42 ± 4^§^	+17.4 ± 11.0	0.007**	0.013*	0.018*
LVIDd (cm)	4.9 ± 0.1	5.0 ± 0.2	+2.1 ± 3.7	0.557	0.446	0.683
LV systolic parameters						
LVOT VTI (cm)	19.9 ± 0.9^§^	21.3 ± 1.1	+6.7 ± 5.4	0.062	0.025*	0.064
LVSV (mL)	59 ± 6^§^	67 ± 5^§^	+12.3 ± 8.2	0.011*	0.024*	0.031*
Mechdisp (ms)	29 ± 3^#^	27 ± 7^‡^	−7.3 ± 9.7	0.127	0.141	0.130
Twist rate (°/s)	101.9 ± 11.4	88.2 ± 2.8^§^	−13.5 ± 15.0	0.057	0.071	0.063
LV diastolic parameters						
LA_EmF_ (%)	68.4 ± 2.4	65.8 ± 2.5	−3.8 ± 5.2	0.145	0.173	0.161
MV_A_ (m/s)	0.42 ± 0.03	0.38 ± 0.04^§^	−10.6 ± 8.3	0.045*	0.027*	0.060
MV_D‐slope_ (m/s^2^)	5.9 ± 0.7	4.9 ± 0.6	−17.1 ± 15.2	0.046*	0.039*	0.053
MV_DT_ (ms)	161 ± 14	178 ± 15	+10.7 ± 10.2	0.046*	0.033*	0.046*
MV_E_ (m/s)	0.9 ± 0.1	0.8 ± 0.1	−7.9 ± 9.3	0.118	0.183	0.160
Untwisting rate (°/s)	−103.6 ± 15.3	−92.1 ± 12.4^§^	−11.1 ± 13.6	0.076	0.088	0.076

*Note:* The table shows the effect of controlling for the change in resting HR, increased BV, and the combination of the two when assessing significance. Values are presented as mean ± 95% confidence interval, *N* = 28.

Abbreviations: LA_EmF_, passive left atrial emptying fraction; LVEDV, left ventricular end‐diastolic volume; LVESV, left ventricular end‐systolic volume; LVIDd, diastolic left ventricular inner diameter; LVOT VTI, left ventricular outflow tract velocity time integral; LVSV, left ventricular stroke volume; Mechdisp, mechanical dispersion; MV_A_, mitral valve A‐wave velocity; MV_D‐slope_, mitral valve deceleration slope; MV_DT_, mitral valve deceleration time; MV_E_, mitral valve E‐wave velocity.

^§^ *N* = 27.

^#^ *N* = 25.

^‡^ *N* = 24.

* *p* < 0.05.

** *p* < 0.01.

Seven echocardiographic values were significantly different between pre‐ and post‐exercise. LV end‐diastolic volume (LVEDV) and LV end‐systolic volume (LVESV) increased by 14.3% (+14 ± 8 mL; *p* = 0.0016) and 17.4% (+6 ± 4 mL; *p* = 0.0032) from baseline, respectively. LVSV and the LV outflow tract velocity time integral (LVOT VTI), also increased from pre to post by 12.3% (+7 ± 5 mL/beat; *p* = 0.0048) and 6.7% (+1.34 ± 1.1 cm; *p* = 0.018), respectively. Lastly, mitral valve A‐wave velocity (MV_A_) decreased by 10.7% (−0.06 ± 0.04 m/s; *p* = 0.014), the mitral valve deceleration slope (MV_D‐slope_) decreased at post by 17.1% (−1.0 ± 0.9 m/s^2^; *p* = 0.029), while the MV_DT_ increased by 10.7% (+17 ± 16 ms; *p* = 0.041).

Six of these variables remained significantly different between pre and post when controlling for the reduction in resting HR. The increase in LVEDV (*p* = 0.004), LVESV (*p* = 0.007), LVSV (*p* = 0.011), and MV_DT_ (*p* = 0.046) as well as the decrease in MV_A_ (*p* = 0.045) and MV_D‐slope_ (*p* = 0.046) remained significant, while no significant difference in LVOT VTI was detected. Furthermore, when controlling for the combination of HR reduction and increased BV the change in four of the variables remained significant: LVEDV (*p* = 0.013), LVESV (*p* = 0.018), LVSV (*p* = 0.031), and MV_DT_ (*p* = 0.046) (Table 2). No significant differences in delta changes of LV volumes (LVEDV, *p* = 0.306; LVESV, *p* = 0.220; LVSV, *p* = 0.497) were detected between men and women.

## 4 Discussion

A marked increase in V̇O_2max_ was observed paralleled by a concomitant increase in BV and a significant reduction in resting HR. In line with previous research, multivariate analysis of echocardiography data in the present study indicated significantly increased stroke volume (LVSV, LVOT VTI), LV volume (LVEDV, LVESV), systolic (twist rate, mechanical dispersion), and diastolic function (MV_A_, MV_E_, MV_D‐slope_, MV_DT_, LA emptying fraction, untwisting rate). Importantly, when controlling for the change in BV and HR, the volumetric changes of the LV and the increase in LVSV were larger than what could be accounted for solely by the hypervolemic response and decrease in resting HR following SIT.

The literature is rich in cross‐sectional studies on the cardiac characteristics of endurance athletes compared to sedentary controls, but longitudinal studies on cardiac adaptations are less numerous. The hemodynamic changes, that is, increased preload and afterload, that occur during TET have been considered the primary stimulus for cardiac remodeling during TET [24]. Studies on the effects of SIT on cardiac remodeling are more limited, perhaps due to the assumption that it induces less hemodynamic stress during exercise and therefore should be less comparable to the well‐documented changes during TET, and that the benefit induced by SIT is more related to peripheral factors. Moreover, no previous study has examined the cardiac adaptations associated with the hypervolemic response, either alone or in combination with the reduction in HR, during exercise. Previously, we [25] and others [26, 27, 28, 29] have shown that BV is an important mediator of improvements in V̇O_2max_ after SIT and TET. In addition, changes in BV directly affect hemodynamic components such as cardiac filling pressure [16, 30], LVSV [31], and HR [18]. Convertino [18] has elegantly shown that PV expansion and HR reduction are directly related. Therefore, we hypothesized that the exercise‐induced changes in cardiac output parameters would correlate with the expected increase in BV and decrease in HR.

Both Matsuo et al. [9] and O'Driscoll et al. [10] demonstrated a reduction in resting HR after various SIT protocols, accompanied by measurements of improved LV systolic function at rest. In contrast to our results, the shorter 2‐week intervention by O'Driscoll et al. [10] did not result in a change in LV dimensions. Instead, they reported changes in diastolic parameters such as mitral valve E‐wave velocity and E/e' ratio, suggesting an exercise‐induced improvement in diastolic function. This is consistent with the initial analysis of the present study, in which several indices of systolic and diastolic function at rest were altered after a 6‐week SIT intervention. However, when considering the changes in BV and HR, our analysis shows that several of these changes are driven by BV expansion and a lower HR at rest, which is illustrated by the discrepancy between our uncorrected echocardiographic data and the data corrected for the exercise‐induced hypervolemic response. Nonetheless, our finding that the increase in LVEDV was greater than what could be explained by changes in BV or HR complements previous research and suggests a volumetric remodeling of the heart that goes beyond a mere increase in diastolic filling that could be observed following 6 weeks of interval training. The occurrence of such a BV‐ and HR‐independent mechanism is supported by previous findings that LV volume is preserved despite a reduction in BV after discontinued training [32].

Indeed, a SIT study conducted for only 2 weeks showed an increase in LVEDV and LVESV [8], but no changes in resting HR were reported at this short duration, and the increase in V̇O_2max_ was modest. Thus, the discrepancy between studies in terms of echocardiographic measurements could be related to the duration of the intervention, including the magnitude of the hypervolemic response and the associated decrease in HR, although an influence of baseline cardiac size or function or differences in training volume and exercise protocol cannot be ruled out.

The finding that 6 weeks of SIT had significant effects on LV volume rather than on measures of diastolic function after controlling for BV expansion and reduction in HR at rest was surprising. One might have expected that the improvement in LV relaxation would precede a morphologic change in the ventricle [5], which a shorter intervention or a mid‐intervention assessment possibly could have shown. However, it is important to note that LVEDV and LVSV increased by ~10% with no evidence of an increase in filling pressures or restrictive filling patterns. This indicates an improvement in diastolic function compared to the active remodeling process, in which the slope of the relationship between LVEDV and filling pressure has changed. There was a BV‐ and HR‐independent increase in MV_DT_ after exercise which suggests a slower passive equalization of pressure between LA and LV during diastole, but in the absence of any change in other diastolic parameters, it is not possible to even speculate whether left atrial pressure (LAP) or LV end‐diastolic pressure (LVEDP) is higher or lower than before exercise. Further studies are needed to reveal the diastolic effects of SIT.

An important focus of research to date has been the exercise effects on the hypertrophic aspects of cardiac remodeling. Cross‐sectional data have shown that the increased LV cavity dimensions are accompanied by a proportional 15%–20% increase in LV wall thickness among endurance athletes compared to controls [1, 33, 34], which, according to La Place's law, allows the wall tension to remain unchanged despite an increased chamber diameter. However, there are contradictory results throughout the literature. Namely, Arbab‐Zadeh et al. [35] demonstrated an initial concentric hypertrophic response in previously sedentary participants during the first 6–9 months of endurance training, while Weiner et al. [36] demonstrated that, during the first 3 months of training, the increase in calculated LV mass was solely attributed to the concomitant increase in LVEDV. In line with Weiner et al., we found no increase in calculated LV mass, interventricular septal or lateral wall thickness in the present study. There are several potential explanations for the difference between the initiation of cardiac remodeling, it can be due to either the length of the intervention, where increased diastolic filling has not yet led to a compensatory hypertrophy, differences in exercise mode, intensity and hypervolemic response or simply intrinsic limitations in the echocardiographic assessment of LV mass.

There are some limitations to be considered in our study. First, we did not include a control group nor was a cross‐over design applied, limiting our ability to control for potential confounding factors resulting in preload differences, even though no systematic effects of time should be expected in healthy subjects after only a few weeks [10, 37]. Second, variables derived using echocardiography generally have high interobserver variability, where variation upward of 15%–24% have been demonstrated [38, 39]. Considering this large variation in relation to the effect sizes observed in our study, we attempt to measure relatively small changes. To minimize this variability, only one operator analyzed each echocardiographic measurement, blinded to pre‐ or post‐intervention and a relatively large study group was included. We have also previously reported low intra‐ and interobserver variability for both linear and volumetric LV measures at our lab [40]. Additionally, echocardiographic exams were performed at rest, preventing us from detecting potential cardiac adaptations present during exercise. Lastly, an experimental design in which hypervolemia is removed through phlebotomy would provide a more detailed understanding when addressing the effects of exercise‐induced hypervolemia on cardiac adaptations.

### 4.1 Perspective

In recent years, it has been evident that the central adaptation of BV expansion is an important mediator of improvements in V̇O_2max_ after both SIT and TET [12, 14, 25]. Exercise training has also been shown to lead to changes in cardiac structure [5]. However, it is not known whether this is driven by passive filling due to BV expansion or actual structural remodeling. Here, we demonstrate that both total circulating BV and LV volumes increase significantly following SIT, but that the increase in LV volume is proportionally greater than the changes in both BV and HR. This indicates that the volumetric remodeling of the heart goes beyond a mere increase in diastolic filling, suggesting intrinsic cardiac adaptations even after a short period of regular SIT. This is of clinical significance for cardiac diastolic function, as the estimated filling pressure was not altered despite the increase in venous return. These results are indicative of the importance of both increased BV and cardiac adaptations for the increase in cardiac output, and consequently, improving V̇O_2max_ following exercise.

### 4.2 Conclusions

In summary, our data suggest that several measures of cardiac function are probably only indirectly altered by SIT due to increased BV. However, the size of the left ventricle increases disproportionately to this change, indicating volume‐independent cardiac remodeling.

## Conflicts of Interest

The authors declare no conflicts of interest.

## Supporting information


Figure S1.

## Data Availability

The data that support the findings of this study are available from the corresponding author upon reasonable request.

## References

[1] V. Utomi , D. Oxborough , G. P. Whyte , et al., “Systematic Review and Meta‐Analysis of Training Mode, Imaging Modality and Body Size Influences on the Morphology and Function of the Male Athlete's Heart,” Heart 99, no. 23 (2013): 1727–1733. 23474689 10.1136/heartjnl-2012-303465

[2] W. R. Roeske , R. A. O'Rourke , A. Klein , G. Leopold , and J. S. Karliner , “Noninvasive Evaluation of Ventricular Hypertrophy in Professional Athletes,” Circulation 53, no. 2 (1976): 286–291. 128424 10.1161/01.cir.53.2.286

[3] A. D'Andrea , L. Riegler , R. Cocchia , et al., “Left Atrial Volume Index in Highly Trained Athletes,” American Heart Journal 159, no. 6 (2010): 1155–1161. 20569734 10.1016/j.ahj.2010.03.036

[4] A. Urhausen , T. Monz , and W. Kindermann , “Sports‐Specific Adaptation of Left Ventricular Muscle Mass in Athlete's Heart. II: An Echocardiographic Study With 400‐m Runners and Soccer Players,” International Journal of Sports Medicine 17, no. Suppl 3 (1996): S152–S156. 9119536 10.1055/s-2007-972917

[5] Y. Hellsten and M. Nyberg , “Cardiovascular Adaptations to Exercise Training,” Comprehensive Physiology 6 (2016): 1–32. 10.1002/cphy.c140080 26756625

[6] S. Caselli , F. M. Di Paolo , C. Pisicchio , N. G. Pandian , and A. Pelliccia , “Patterns of Left Ventricular Diastolic Function in Olympic Athletes,” Journal of the American Society of Echocardiography 28, no. 2 (2015): 236–244. 25441331 10.1016/j.echo.2014.09.013

[7] A. Arbab‐Zadeh , E. Dijk , A. Prasad , et al., “Effect of Aging and Physical Activity on Left Ventricular Compliance,” Circulation 110, no. 13 (2004): 1799–1805. 15364801 10.1161/01.CIR.0000142863.71285.74

[8] J.‐J. Eskelinen , I. Heinonen , E. Löyttyniemi , et al., “Left Ventricular Vascular and Metabolic Adaptations to High‐Intensity Interval and Moderate Intensity Continuous Training: A Randomized Trial in Healthy Middle‐Aged Men,” Journal of Physiology 594, no. 23 (2016): 7127–7140. 27500951 10.1113/JP273089 PMC5134384

[9] T. Matsuo , K. Saotome , S. Seino , et al., “Effects of a Low‐Volume Aerobic‐Type Interval Exercise on V̇O_2_max and Cardiac Mass,” Medicine and Science in Sports and Exercise 46, no. 1 (2014): 42–50. 23846165 10.1249/MSS.0b013e3182a38da8

[10] J. M. O'Driscoll , S. M. Wright , K. A. Taylor , D. A. Coleman , R. Sharma , and J. D. Wiles , “Cardiac Autonomic and Left Ventricular Mechanics Following High Intensity Interval Training: A Randomized Crossover Controlled Study,” Journal of Applied Physiology 125, no. 4 (2018): 1030–1040. 29952247 10.1152/japplphysiol.00056.2018 PMC6230570

[11] J. Morganroth , B. J. Maron , W. L. Henry , and S. E. Epstein , “Comparative Left Ventricular Dimensions in Trained Athletes,” Annals of Internal Medicine 82, no. 4 (1975): 521–524. 1119766 10.7326/0003-4819-82-4-521

[12] D. Montero and C. Lundby , “Red Cell Volume Response to Exercise Training: Association With Aging,” Scandinavian Journal of Medicine & Science in Sports 27, no. 7 (2017): 674–683. 27859711 10.1111/sms.12798

[13] M. Mandić , B. Hansson , A. Lovrić , et al., “Improvements in Maximal Oxygen Uptake After Sprint‐Interval Training Coincide With Increases in Central Hemodynamic Factors,” Medicine and Science in Sports and Exercise 54, no. 6 (2022): 944–952. 35136000 10.1249/MSS.0000000000002872

[14] J. Schierbauer , T. Hoffmeister , G. Treff , N. B. Wachsmuth , and W. F. J. Schmidt , “Effect of Exercise‐Induced Reductions in Blood Volume on Cardiac Output and Oxygen Transport Capacity,” Frontiers in Physiology 12 (2021): 679232. 34135772 10.3389/fphys.2021.679232 PMC8201095

[15] D. E. R. Warburton , M. J. Haykowsky , H. A. Quinney , et al., “Blood Volume Expansion and Cardiorespiratory Function: Effects of Training Modality,” Medicine and Science in Sports and Exercise 36, no. 6 (2004): 991–1000. 15179169 10.1249/01.mss.0000128163.88298.cb

[16] V. A. Convertino , G. W. Mack , and E. R. Nadel , “Elevated Central Venous Pressure: A Consequence of Exercise Training‐Induced Hypervolemia?,” American Journal of Physiology 260, no. 2 Pt 2 (1991): R273–R277. 1996713 10.1152/ajpregu.1991.260.2.R273

[17] M. L. Smith , D. L. Hudson , H. M. Graitzer , and P. B. Raven , “Exercise Training Bradycardia: The Role of Autonomic Balance,” Medicine and Science in Sports and Exercise 21, no. 1 (1989): 40–44. 2927300 10.1249/00005768-198902000-00008

[18] V. A. Convertino , “Heart Rate and Sweat Rate Responses Associated With Exercise‐Induced Hypervolemia,” Medicine and Science in Sports and Exercise 15, no. 1 (1983): 77–82. 6843324

[19] S. Keiser , A.‐K. Meinild‐Lundby , T. Steiner , et al., “Detection of Blood Volumes and Haemoglobin Mass by Means of CO Re‐Breathing and Indocyanine Green and Sodium Fluorescein Injections,” Scandinavian Journal of Clinical and Laboratory Investigation 77, no. 3 (2017): 164–174. 28276723 10.1080/00365513.2016.1271908

[20] N. Prommer and W. Schmidt , “Loss of CO From the Intravascular Bed and Its Impact on the Optimised CO‐Rebreathing Method,” European Journal of Applied Physiology 100, no. 4 (2007): 383–391. 17394011 10.1007/s00421-007-0439-2

[21] C. J. Gore , W. G. Hopkins , and C. M. Burge , “Errors of Measurement for Blood Volume Parameters: A Meta‐Analysis,” Journal of Applied Physiology 99, no. 5 (2005): 1745–1758. 15976358 10.1152/japplphysiol.00505.2005

[22] R. M. Lang , L. P. Badano , V. Mor‐Avi , et al., “Recommendations for Cardiac Chamber Quantification by Echocardiography in Adults: An Update From the American Society of Echocardiography and the European Association of Cardiovascular Imaging,” Journal of the American Society of Echocardiography 28, no. 1 (2015): 1–39.e14. 25559473 10.1016/j.echo.2014.10.003

[23] S. F. Nagueh , O. A. Smiseth , C. P. Appleton , et al., “Recommendations for the Evaluation of Left Ventricular Diastolic Function by Echocardiography: An Update From the American Society of Echocardiography and the European Association of Cardiovascular Imaging,” Journal of the American Society of Echocardiography 29, no. 4 (2016): 277–314. 27037982 10.1016/j.echo.2016.01.011

[24] A. N. Kavazis , “Pathological vs. Physiological Cardiac Hypertrophy,” Journal of Physiology 593, no. Pt 17 (2015): 3767. 26331830 10.1113/JP271161 PMC4575564

[25] M. Mandić , L. M. J. Eriksson , M. Melin , et al., “Increased Maximal Oxygen Uptake After Sprint‐Interval Training Is Mediated by Central Haemodynamic Factors as Determined by Right Heart Catheterization,” Journal of Physiology 601, no. 12 (2023): 2359–2370. 37071120 10.1113/JP283807

[26] V. A. Convertino , “Blood Volume Response to Physical Activity and Inactivity,” American Journal of the Medical Sciences 334, no. 1 (2007): 72–79. 17630597 10.1097/MAJ.0b013e318063c6e4

[27] T. C. Bonne , G. Doucende , D. Flück , et al., “Phlebotomy Eliminates the Maximal Cardiac Output Response to Six Weeks of Exercise Training,” American Journal of Physiology‐Regulatory, Integrative and Comparative Physiology 306, no. 10 (2014): R752–R760. 24622974 10.1152/ajpregu.00028.2014

[28] D. Montero , A. Cathomen , R. A. Jacobs , et al., “Haematological Rather Than Skeletal Muscle Adaptations Contribute to the Increase in Peak Oxygen Uptake Induced by Moderate Endurance Training,” Journal of Physiology 593, no. 20 (2015): 4677–4688. 26282186 10.1113/JP270250 PMC4606528

[29] Ø. Skattebo , E. S. Johansen , C. Capelli , and J. Hallén , “Effects of 150‐ and 450‐mL Acute Blood Losses on Maximal Oxygen Uptake and Exercise Capacity,” Medicine and Science in Sports and Exercise 53, no. 8 (2021): 1729–1738. 34261996 10.1249/MSS.0000000000002618

[30] A. C. Guyton , A. W. Lindsey , B. Abernathy , and T. Richardson , “Venous Return at Various Right Atrial Pressures and the Normal Venous Return Curve,” American Journal of Physiology 189, no. 3 (1957): 609–615. 13458395 10.1152/ajplegacy.1957.189.3.609

[31] M. K. Hopper , A. R. Coggan , and E. F. Coyle , “Exercise Stroke Volume Relative to Plasma‐Volume Expansion,” Journal of Applied Physiology 64, no. 1 (1988): 404–408. 2451658 10.1152/jappl.1988.64.1.404

[32] E. M. Cullinane , S. P. Sady , L. Vadeboncoeur , M. Burke , and P. D. Thompson , “Cardiac Size and VO_2_max Do Not Decrease After Short‐Term Exercise Cessation,” Medicine and Science in Sports and Exercise 18, no. 4 (1986): 420–424. 3747802

[33] B. J. Maron , “Structural Features of the Athlete Heart as Defined by Echocardiography,” Journal of the American College of Cardiology 7, no. 1 (1986): 190–203. 2934463 10.1016/s0735-1097(86)80282-0

[34] T. Venckunas , R. Raugaliene , B. Mazutaitiene , and S. Ramoskeviciute , “Endurance Rather Than Sprint Running Training Increases Left Ventricular Wall Thickness in Female Athletes,” European Journal of Applied Physiology 102, no. 3 (2008): 307–311. 17940792 10.1007/s00421-007-0586-5

[35] A. Arbab‐Zadeh , M. Perhonen , E. Howden , et al., “Cardiac Remodeling in Response to 1 Year of Intensive Endurance Training,” Circulation 130, no. 24 (2014): 2152–2161. 25281664 10.1161/CIRCULATIONAHA.114.010775 PMC5698012

[36] R. B. Weiner , J. R. DeLuca , F. Wang , et al., “Exercise‐Induced Left Ventricular Remodeling Among Competitive Athletes,” Circulation. Cardiovascular Imaging 8, no. 12 (2015): e003651. 26666381 10.1161/CIRCIMAGING.115.003651

[37] M. Scharf , A. Schmid , W. Kemmler , et al., “Myocardial Adaptation to High‐Intensity (Interval) Training in Previously Untrained Men With a Longitudinal Cardiovascular Magnetic Resonance Imaging Study (Running Study and Heart Trial),” Circulation. Cardiovascular Imaging 8, no. 4 (2015): e002566. 25873721 10.1161/CIRCIMAGING.114.002566

[38] S. Malm , S. Frigstad , E. Sagberg , H. Larsson , and T. Skjaerpe , “Accurate and Reproducible Measurement of Left Ventricular Volume and Ejection Fraction by Contrast Echocardiography: A Comparison With Magnetic Resonance Imaging,” Journal of the American College of Cardiology 44, no. 5 (2004): 1030–1035. 15337215 10.1016/j.jacc.2004.05.068

[39] L. D. Jacobs , I. S. Salgo , S. Goonewardena , et al., “Rapid Online Quantification of Left Ventricular Volume From Real‐Time Three‐Dimensional Echocardiographic Data,” European Heart Journal 27, no. 4 (2006): 460–468. 16319085 10.1093/eurheartj/ehi666

[40] K. Hedman , É. Tamás , J. Henriksson , N. Bjarnegård , L. Brudin , and E. Nylander , “Female Athlete's Heart: Systolic and Diastolic Function Related to Circulatory Dimensions,” Scandinavian Journal of Medicine & Science in Sports 25, no. 3 (2015): 372–381. 24840312 10.1111/sms.12246

