# Supplementary material for: Evidence of Left Ventricular Cardiac Remodeling After 6 Weeks of Sprint Interval Training
Source: Scand J Med Sci Sports. 2024 Dec 20;34(12):e70007. doi: 10.1111/sms.70007 (PMC11662151; doi:10.1111/sms.70007)
Supplement: Supplementary file 1 — Figure S1. [file SMS-34-e70007-s001.docx]

**Supplemental Material**

**Supplemental Figure 1**: Principal component analysis was performed on the separate classification groups where the first two principal components accounted for 98% of the variability of variables describing LV morphological features (A), 50% of the variability of variables describing LV systolic function (B), 41% of the variability of variables describing LV diastolic function (C) and 86% of the variability of variables describing RV structure and function, respectively (D). Based on data from all study participants (N = 28). Abbreviations: MV_A_, mitral valve A-wave velocity; Mechdisp, mechanical dispersion; LA_EmF_, passive left atrial emptying fraction; LVESV, left ventricular end systolic volume; LVEDV, left ventricular end diastolic volume; LVSV, left ventricular stroke volume; LVOT VTI, left ventricular outflow tract velocity time integral; MV_D-slope_, mitral valve deceleration slope; LVIDd, diastolic left ventricular inner diameter; MV_DT_, mitral valve deceleration time; MV_E_, mitral valve E-wave velocity; GLS_AvgEpi_, average epicardial global longitudinal strain; GLS_Avg,_, average global longitudinal strain; LVEF, left ventricular ejection fraction; é_RV_, right ventricular free wall é, GLS_AvgEndo_, average endocardial global longitudinal strain; á_RV_, right ventricular free wall á; é_Lat_, left ventricular lateral wall é; TAPSE, tricuspid annular plane systolic excursion; á_Lat_, left ventricular lateral wall á; E/é, mitral e-wave velocity over average left ventricular é; s´_Lat_, left ventricular lateral wall s´; s´_Sept_, left ventricular septal wall s´; é_Sept_, left ventricular septal wall é; s´_RV_, right ventricular free wall s´; á_Sept_, left ventricular septal wall á; RVOT VTI, right ventricular outflow tract velocity time integral; RVID1_(basal)_, right ventricular basal inner diameter.
